# Conformational changes in myeloperoxidase induced by ubiquitin and NETs containing free ISG15 from systemic lupus erythematosus patients promote a pro-inflammatory cytokine response in CD4^+^ T cells

**DOI:** 10.1186/s12967-020-02604-5

**Published:** 2020-11-11

**Authors:** Daniel Alberto Carrillo-Vázquez, Eduardo Jardón-Valadez, Jiram Torres-Ruiz, Guillermo Juárez-Vega, José Luis Maravillas-Montero, David Eduardo Meza-Sánchez, María Lilia Domínguez-López, Jorge Carlos Alcocer Varela, Diana Gómez-Martín

**Affiliations:** 1 Department of Internal Medicine, Instituto Nacional de Ciencias Médicas y Nutrición Salvador Zubirán, Vasco de Quiroga 15, Tlalpan, 14080 Mexico City, Mexico; 2 Department of Immunology and Rheumatology, Instituto Nacional de Ciencias Médicas y Nutrición Salvador Zubirán, Vasco de Quiroga 15, Tlalpan, 14080 Mexico City, Mexico; 3 Department of Immunology, Escuela Nacional de Ciencias Biológicas, Instituto Politécnico Nacional, Ciudad de México, Mexico; 4 Earth Resources Department, Universidad Autónoma Metropolitana, 52005 Lerma, Estado de Mexico Mexico; 5 Emergency Medicine Department, Instituto Nacional de Ciencias Médicas y Nutrición Salvador Zubirán, Vasco de Quiroga 15, Tlalpan, 14080 Mexico City, Mexico; 6 Red de Apoyo a La Investigación, Coordinación de Investigación Científica, Universidad Nacional Autónoma de México, Mexico City, Mexico

**Keywords:** NETs, Post-translational modifications, Myeloperoxidase, Ubiquitin, ISG15, Lupus

## Abstract

**Background:**

Neutrophil extracellular traps (NETs) from patients with systemic lupus erythematosus (SLE) are characterized by lower ubiquitylation and myeloperoxidase (MPO) as a substrate. The structural and functional effect of such modification and if there are additional post-translational modifications (PTMs) are unknown.

**Methods:**

To assess the expression and functional role of PTMs in NETs of patients with SLE; reactivation, proliferation and cytokine production was evaluated by flow cytometry using co-cultures with dendritic cells (DC) and CD4^+^ from SLE patients and healthy controls. The impact of ubiquitylation on MPO was assessed by molecular dynamics. The expression of ISG15 in NETs was evaluated by immunofluorescence and Western Blot.

**Results:**

Fifteen patients with SLE and ten healthy controls were included. In the co-cultures of CD4^+^ lymphocytes with DC stimulated with ubiquitylated MPO or recombinant MPO, a higher expression of IFNγ and IL-17A was found in CD4^+^ from SLE patients (p < 0.05). Furthermore, with DC stimulated with ubiquitylated MPO a trend towards increased expression of CD25 and Ki67 was found in lupus CD4^+^ lymphocytes, while the opposite was documented in controls (p < 0.05). Through molecular dynamics we found the K129-K488-K505 residues of MPO as susceptible to ubiquitylation. Ubiquitylation affects the hydration status of the HEME group depending on the residue to which it is conjugated. R239 was found near by the HEME group when the ubiquitin was in K488-K505. In addition, we found greater expression of ISG15 in the SLE NETs vs controls (p < 0.05), colocalization with H2B (r = 0.81) only in SLE samples and increased production of IFNγ in PBMCs stimulated with lupus NETs compared to healthy controls NETs.

**Conclusion:**

The ubiquitylated MPO has a differential effect on the induction of reactivation of CD4^+^ lymphocytes in patients with SLE, which may be related to structural changes by ubiquitylation at the catalytic site of MPO. Besides a lower ubiquitylation pattern, NETs of patients with SLE are characterized by the expression of ISG15, and the induction of IFNγ by Th1 cells.

## Introduction

Neutrophil extracellular traps (NETs) play a pathogenic role in diverse autoimmune diseases, including systemic lupus erythematosus (SLE) [1]. These NETs are fibrillar mesh structures decorated by nuclear chromatin and neutrophil granular peptides (i.e. myeloperoxidase, elastase, lactoferrin and LL-37) and are one of the most potent tools to combat microorganisms [2]. Likewise, these structures are a potential source of autoantigens, whose externalization might lead to self-tolerance breakdown. Indeed, enhanced NETosis, diminished clearance or post-translational modifications in the protein cargo of these NETs are related to tissue damage in SLE [3]. Diverse stimuli have been acknowledged, including those related to microorganisms, such as LPS, inflammatory stimuli (ie ROS, TNF-α and IL-8) as well as those considered as sterile, such as the antigen–antibody complexes [4]. Indeed, Petretto A, et al. [4] showed by proteomic analysis a differential protein cargo and post-translational modifications, dependent upon the induction stimuli for NETosis; interestingly the majority of modified peptides are derived from myeloperoxidase (MPO), which might be responsible for diverse biologic effects [4]. This enzyme catalyzes the transformation of hydrogen peroxide in hypochlorous acid, hence MPO has a bactericidal function. MPO is key for the NETosis induction, since it acts synergistically with neutrophil elastase (NE) for the degradation of membranes and chromatin decondensation [5]. Furthermore, MPO has been acknowledged as a dual modulator of immune responses, particularly when it is located at the extracellular milieu, where it is able to induce tissue damage mediated by increased oxidative stress [6]. At the cellular level, MPO induces T cell proliferation in a dose dependent manner in antineutrophil cytoplasmic antibodies (ANCA) associated vasculitis and healthy donors [7], as well as having an immunosuppressive role, mediated by IL-10 [8] and by dendritic cell suppression through reduced CD86 and IL-12 expression (6).

Post-translational modifications (PTMs) have been related to NETosis induction and its pathogenic role in autoimmune diseases has been acknowledged, since it implies a mechanism for neoantigen expression. Indeed, citrullination of histone 2B during NETosis is a key element for pathogenic autoantibody responses in SLE [9]. Furthermore, NETs from SLE patients are characterized by a differential PTM profile, including histone hyperacetylation [10] and a deficient polyubiquitilation (K48/K63) profile [11], which induce pro-inflammatory macrophage responses. Among PTMs with potential regulatory role in autoimmune pathogenic responses, the ISGylation, defined as the conjugation of the ubiquitin-like-protein ISG15; is related to enhanced type I IFN responses [12]. Although some of the ISG15 potential targets are inducible by interferogenic stimulation such as the antiviral proteins HuP56, MxA and RIG-I; constitutive substrates for instance maspin, thioredoxin, HSP60 and moesin, have shown conjugation with ISG15 and up to more than one third of them are derived from the nucleus [13]. Besides that, extracellular ISG15 also promotes the synthesis of IFNγ by T and B cells. Additionally, NETs are potent inducers of IFNα/β by plasmacytoid dendritic cells in SLE [14] and type I IFN responses are the hallmark of the molecular signature in SLE. Nonetheless, it has not been addressed if ISG15 is present in NETs from SLE patients as a potential source of ISG15 and a regulator of IFN responses.

Evidence suggests that PTMs, particularly ubiquitylation and ISGylation might play a role for enhanced NETosis in SLE and particularly, MPO was shown to be target of this PTM in NETs from SLE patients and healthy donors [11]. However, the precise amino acid residue where ubiquitin tag is added is currently unknown, as well as its potential impact in cellular responses. Therefore, the aim of the present work is to address the role of PTMs related to ubiquitin and ubiquitin-like-protein, ISG15 from NETs and their effects in the regulation of cellular responses in SLE.

## Materials and methods

**Patients and controls:** We recruited 15 Mexican-mestizo adult patients with active SLE (SLEDAI > 6) according to the ACR criteria [15], who were followed-up in a tertiary care center (Instituto Nacional de Ciencias Médicas y Nutrición Salvador Zubirán). As a control group, ten age and gender matched healthy subjects were included. Exclusion criteria were applied to those patients with any kind of acute or chronic infection, pregnancy, puerperium and neoplasia. All healthy controls and SLE patients signed an informed consent before inclusion, and the protocol was approved by our Institutional Ethics Committee (Ref. 2152) in compliance with the Helsinki declaration. Laboratory features of SLE patients were assessed as follows: Auto antibodies of SLE patients (anti-dsDNA IgG, anti-nucleaosomes, anti-cardiolipin and anti-β2glycoprotein I) were assayed using a commercial ELISA.

**In vitro ubiquitylation of MPO:** A commercial *Ubiquitylation Kit (Abcam™)* (HeLa lysate) was used according to the manufacturer’s instructions. The recombinant human MPO (rhMPO 10 µg) was incubated for 4 h at 37° in a water bath and stored at − 80°. To confirm that the ubiquitylation reaction took place, a 4–15% polyacrylamide gel electrophoresis was performed with 50 µg of ubiquitylated MPO (UbMPO) and rhMPO. Subsequently, the transfer to a PVDF membrane was carried out for 1 h at 100v, then, the non-specific binding sites were blocked with Starting Block solution (*Thermo Fisher ™*). Overnight incubation was carried out with the primary anti-ubiquitin antibody (P4D1 *Santa Cruz™*) and followed by the secondary antibody HRP anti-Mouse for 1 h (*Thermo Fisher™*). Membranes were developed by enhanced chemiluminiscence with ECL Western Blotting Substrate (*BioRad™*). Samples were acquired with a digital image analyzer (*Chemidoc MP™, Biorad™*) and quantified by densitometry with ImageLab software (*Biorad™*). (Additional file 1: Figure S1, Panel A).

**T cell responses upon ubiquitylated MPO (UbMPO):** To address T cell responses, cocultures of mature in vitro CD4^+^ T lymphocytes and monocyte derived dendritic cells (MoDCs) were set up and stimulated with either the native MPO (recombinant human MPO, rhMPO) (5 μg/ml) or UbMPO (5 μg/ml).

**Monocyte isolation and MoDCs generation:** Peripheral blood mononuclear cells (PBMCs) were isolated from SLE patients and healthy controls by density gradients after centrifugation with *Ficoll-Paque PLUS (GE Healthcare™*). After washing twice with 5% fetal bovine serum (FBS) in PBS, PBMCs were resuspended in RPMI 1640 medium (*Gibco*™) supplemented with 10% FBS and 100,000 cells were incubated for 30 min at 4 °C in MACS buffer and anti-CD14 antibodies from CD14 MicroBeads Isolation Kit conjugated with magnetic beads (*Miltenyi Biotec* ™) to obtain the enriched CD14^+^ population. To induce differentiation to dendritic cells, GM-CSF (*R&D*™) (50 ng/ml) and IL-4 (*PeProTech*™) (30 ng/ml) were added. CD14^+^ monocytes were seeded in 6-well plates in 4 ml (1 × 10^6^ per ml of medium). On the third day, medium change was performed [16]. After 7 days, DCs were harvested and viability was evaluated using the trypan blue method. They were seeded in 24-well plates (1 × 10^6^ cells per ml of medium). Cells were divided into three reactivation conditions for 24 h [17]: (a) LPS (1 μg/ml) *E. coli* O111: B4 LPS (*Sigma Aldrich™)*; (b) LPS (1 μg/ml) and rhMPO R&D™ (5 μg/ml); and (c) LPS (1 μg/ml) and ubiquitylated MPO (5 μg/ml).

**CD4**^**+**^ **T cell isolation and CD4**^**+**^**/MoDC cocultures**: Both lupus and control CD4^+^ T cells were also isolated from total PBMCs by magnetic negative selection with microbeads according to the Human CD4^+^ Isolation Kit manufacturer instructions (*Miltenyi Biotec™)*. CD4^+^/MoDC cocultures were performed in a 10:1 ratio at 37 °C, and 4% CO_2_ for 3 days to evaluate proliferation and for 7 days to evaluate reactivation and cytokine production from CD4^+^ T cells. The cocultures were set up as follows: (a) Unstimulated CD4^+^ cells; (b) CD4^+^ cells plus DCs activated only with LPS; (c) CD4^+^ cells plus DCs activated with LPS and rhMPO; (d) CD4^+^ cells plus DCs activated with LPS and ubiquitylated MPO; and (e) CD4^+^ cells polyclonally stimulated with 50 ng/mL of αCD3 (*HIT3a BD™*) and 100 ng/mL of αCD28 (*NA/LE BD™).*

**CD4**^**+**^ **T cell proliferation and activation markers assessment by flow cytometry:** For the assessment of activation markers, after washing twice with 5% FBS in PBS and incubated with FVS700 to evaluate viability, PBMCs were stained with the following fluorescent surface labelled-antibodies: (CD4-APC, CD25-PE, CD83-PE/Cy5, HLA-DR-PerCP) *(*All *Bio Legend™)*. After that, the cells were fixed with *True-Nuclear™ 1X Fix Concentrate,* then washed with *FoxP3-Perm Buffer™* and finally incubated with labelled anti-FoxP3 BV421. For the proliferation assay, the same steps described above were performed and after fixation and permeabilization, PBMCs were incubated with anti Ki67-APC *(Bio Legend™)* instead*.*

For intracellular cytokine staining, PBMCs were stimulated with PMA (*Sigma™*), ionomycin from *Streptomyces conglobatus (Sigma™)* and incubated with monensin (*GolgiStop*™, *BD Horizon™*) at 37ºC for 5 h. After washing twice with 5% FBS in PBS, PBMCs were stained with anti CD4-AF488 and then fixed and permeabilized with *Cytofix/cytoperm (BD™),* and finally incubated with intracellular anti IFNγ-APC (Th1), IL-4-PE (Th2), IL-17A-BV421V (Th17) (*Bio Legend™*) (Additional file 1: Figure S2)*.*

**Molecular dynamics methods:** To assess the initial positions of all-atoms of the backbone of the MPO were taken from the cryogenic crystal structure isoform C, PDB:1CXP, chains A and C [18], while the ubiquitin coordinates were taken from the crystal structure, PDB:1UBQ, chain B [19]. To approach the post-translational modifications of MPO, the solvent accessible surface area (SASA) and the root mean square deviation (RMSD) fit of MPO at lysine residues with K6, K48 and K63 of the ubiquitin, were used as criterium to identify exposed lysines. Larger values of SASA were used to identify susceptible residues for ubiquitylation (an enzymatic reaction that its defined by an isopeptide bond among the amino group of substrate lysine chains and the carboxyl terminus G63 of ubiquitin).

To the generation of simulation trajectories, MPO was defined as the center in the simulation box and the water molecules were added via the solvate plugin of VMD 1.9.1 program [20], filling the space around the protein after a 2 Å cutoff. To define the physiologic electrolyte concentration of NaCl at 0.15 M we used the ionized VMD plugin [20]. Every system was committed to energy minimization for 10 K steps of conjugate gradient algorithm. Later, the crystallographic structure was relaxed in steps of 200 ps each, with positional constraints setting force constants of 25, 15, 10 5 3, 2,1 kcal/mol Å. After pre-equilibration, all simulation trajectories were produced with the NAMD 2.12 program [21]. Configurations in the isothermal-isobaric ensemble, at 300 K and 1 bar conditions, were generated using Langevin dynamics scheme to maintain constant temperature [21], and a Nose–Hoover Langevin piston for pressure control [22, 23]. For computational efficiency, a multiple time-step integration scheme was set, with 1 fs for bond forces, 2 fs for short-range non-bonding interactions, and 4 fs for full electrostatic forces [24, 25]. Coulomb interactions were computed using particle-mesh smooth Ewald summation [26, 27] with a tolerance of 10^–6^ for the direct part of the Ewald sum, a fourth-order interpolation scheme, and a grid spacing of ~ 1 Å, for each direction of the simulation box. All bonds for hydrogen atoms were constrained using the SHAKE algorithm [28]. All-atom force field parameters were used in this study. The CHARMM 36 force field [29–31] with CMAP correction for the protein atoms [32], and the TIP3P water model [33]. In house analysis scripts were developed for specific calculations, such as hydration level at the active site, heme group contact maps.

**ISG15 detection in ex vivo NETs by Western Blot:** After density gradients, neutrophils were isolated with dextran sedimentation as previously described [34]. We assessed the spontaneous NET formation (without stimuli) and lipopolysaccharide (LPS)-induced NETosis with 1 μg/mL *E. coli* O111:B4 LPS (*Sigma Aldrich* ™) for 4 h. Subsequently, 300 μL of RPMI with micrococcal nuclease (0.01U/µL) were added, the supernatant was centrifuged and stored at − 20 °C. The protein was quantified using the bicinchoninic acid method at a wavelength of 562 nm. We storage aliquots for functional experiments, which are described later. 4–15% polyacrylamide gel electrophoresis was performed on the lysates from the NETs of patients with SLE and healthy controls. Subsequently, the transfer was carried out for 1 hour at 100v to a PVDF membrane. Nonspecific binding sites were blocked with the Starting Block (*Thermo Fisher*™) solution. The membrane was then incubated overnight with anti-ISG15 (*Abcam* ™), after 3 washes with TBS-tween, the secondary antibody HRP anti-Rabbit (*Thermo Fisher™*) was added for 1 h. Membranes were developed by enhanced chemiluminiscence with ECL Western Blotting Substrate (*BioRad).* In order to validate the anti-ISG15 antibody, we used 10 μg of lysate transfected human HEK293T cells that overexpressed ISG15 (*Novus™*) as a positive control and 10 μg of an empty vector transfected control cell lysate (HEK293) as a negative control (*Novus™*). We perform a Western Blot of those controls as previously described above (Additional file 1: Figure S1, Panel B). Samples were acquired with a digital image analyzer (*Chemidoc MP, Biorad*) and quantified by densitometry with *ImageLab* software (*Biorad*). Data was expressed as normalized expression to MPO.

**Assessment of ISG15 in** **ex vivo NETs by confocal microscopy:** Neutrophils were incubated in RPMI without phenol red (*Thermo Fisher* ™), 1% FBS, and 1% 10 mM 4-(2-hydroxyethyl)-1-piperazineethanesulfonic acid (HEPES) buffer. For confocal microscopy, neutrophils were seeded in 0.01% poly-L-Lysine (*Sigma-Aldrich ™*) coated coverslips at 37ºC during 1.5 h. After fixation with 4% paraformaldehyde (*Santa Cruz ™*) [34] at 4 °C during 24 h, washing and blocking with 0.2% gelatin from porcine skin (*Sigma-Aldrich* ™) was performed. For indirect immunofluorescence, we used the following primary and secondary antibodies diluted in 0.2% gelatin from porcine skin: rabbit anti-human ISG15 (*Abcam™*), mouse anti-human H2B (*Abcam™*), donkey anti-rabbit Alexa Fluor 555 (*Thermo Fisher™*) and donkey anti-mouse Alexa Fluor 488 (*Thermo Fisher™*). Additionally, we used mouse anti-human LL37 *(LSBio™)* or mouse anti-human HMGB1 (*Thermo Fisher™*). (Additional file 1: Figures S5 and S6). Chromatin was stained with Hoechst 33342 (*Thermo Fisher™*) and coverslips were mounted on slides with ProLong™ Gold Antifade Mountant (*Thermo Fisher™*) [35]. The samples were acquired in an Eclipse Ti-E *Nikon* confocal microscope (Minato, Tokyo, Japan). The number of cells positive for H2B and nuclear staining (Hoechst) were considered producers of NETs. The mean fluorescence intensity and the area percentage of NETs were quantified using Fiji free software (*ImageJ* ™) using the average of 6 fields at 40×, normalized for the total number of cells analyzed. The colocalization of ISG15 with histone 2B (H2B) was calculated by Pearson correlation coefficient and validated through the Costes method [36].

**Flow cytometry assessment of IFNγ production in PBMC's stimulated with NETs containing ISG15:** To evaluate IFNγ production, 2 × 10^6^ healthy donor’s PBMCs were stimulated with 100 μg/mL of SLE NETs containing ISG15 and NETs from healthy controls lacking ISG15 (NETs were induced as previously described in this section, the expression of ISG15 were corroborated by Western Blot and immunofluorescence) or PMA *Sigma*™ (50 ng/mL) and ionomycin from *Streptomyces conglobatus Sigma*™ (1 μg/mL) as a positive control. Monensin (*GolgiStop*™ *BD Horizon*™) were added and incubated at 37 °C for 18 h. After washing twice with 5% FBS in PBS, PBMCs were stained with the following fluorescent surface labelled-antibodies: (CD3-APCH7, CD4-AF488, CD8-PE/Dazzle, CD56-PE, CD335-BV711) (All *Bio Legend™)*. CD4^+^ cells were defined as CD3^+^CD4^+^, CD8^+^ cells were CD3^+^CD8^+^ and NK cells were CD3^−^CD56^+^CD335^+^. After that, the cells were fixed and permeabilized with *Cytofix/cytoperm BD™*, and finally incubated with intracellular anti IFNγ-APC (Th1) (*Bio Legend™*). The samples were acquired on a *BD LSRFortessa* flow cytometer. Data analysis was performed with the support of *FlowJo* 10.6 software (*FlowStar Inc ™*).

**Statistical analysis: **Quantitative variables were expressed as median with interquartile range (IQR) or minimum and maximum (min–max). The homogeneity of variances of each experiment was determined using the Brown-Forsythe and Bartlett tests. The non-parametric data were analyzed with the Kruskall-Wallis test and the adjustment was made using the Dunn multiple comparison test, as well as Mann–Whitney *U* test (sum of Wilcoxon ranges) to compare medians between groups with free software *Past 3* version™ (United States). Pearson's linear correlation coefficient was used [37], and significance was verified using the Costes method [36]. In all cases, significant differences were considered fora p value lower than 0.05. Statistical analysis and graph generation was performed with support of the *GraphPad Prism 8* version ™ software (United States).

## Results

**Demographic and clinical features of patients with SLE:** Table 1 shows the main characteristics of the fifteen patients with active SLE included (SLEDAI > 6), of which 80% were women and 20% men. The median age was 27.18 years (19–35 years). The disease activity was measured using the SLEDAI score [38] with a median of 20 points (8–24), 90.0% had constitutional activity, 82% renal activity, 72.7% hematological activity and 63.6% mucocutaneous activity at the time of the blood draw. All had positive anti-nuclear antibodies in different patterns, 86.6% had anti-dsDNA IgG in positive titers with a median of 142 IU/mL (2.1–485 IU/mL), 33.3% had anti- nucleosomes with a median of 209 U/mL (11.4–660 U/mL). 86% of patients had hypocomplementemia. All patients used immunosuppressive treatment at the time of sampling.

**Table 1:** Laboratory features to evaluate the disease activity and therapeutics used in SLE patients at the time of sampling

Variable	Median (IQR)
*Disease activity*	
SLEDAI	20 (8–24)
*General laboratory features*	
Total leukocyte count (× 10^9^ × L)	5.087 (4–10.1)
Total neutrophil count (× 10^6^ × L)	5087 (3266–7979)
Total lymphocyte count (× 10^6^ × L)	1390 (846–1847)
Neutrophil/lymphocyte index	8 (1–19)
Hemoglobin (g/dL)	10.5 (6.6–13.7)
Creatinine (mg/dL)	1.8 (0.6–5.19)
C3 (mg/dL)	57 (32–110)
C4 (mg/dL)	8 (8–28)
Proteinuria (mg/24 h)	7555.3 (1866–19,564)
Proteinuria-Creatinuria Index (CPI) g/g	5.78 (1.5–14.5)
*Immunological tests*	
Anti-DNAdc IgG (ELISA-FARR) (IU/mL)	141.1 (2.1–658)
Anti-Nucleosomes (U/mL)	209 (11.4–659.7)
IgM anti-cardiolipin (UMPL)	10.9 (7.3–33.8)
Anti-cardiolipin IgG (UGPL)	8.95 (4.7–65.3)
Anti-β2glycoprotein I IgM (U/mL)	4.3 (3–14.2)
Anti-β2glycoprotein I IgG (U/mL)	4.05 (2.8–72.5)
Positive lupus anticoagulant	20%
*Immunosuppressive therapy*	
Prednisone dose (mg/day)	51.8 (10–70)
Azathioprine dose (mg/day)	1.25 (1.25–1.25)
Mycophenolic acid dose (gr/day)	3 (2.5–3)
Cyclophosphamide dose (gr/month)	1 (0.8–1.4)
Dose of hydroxychloroquine (mg/day)	200 (150–400)

**Fig. 1:**
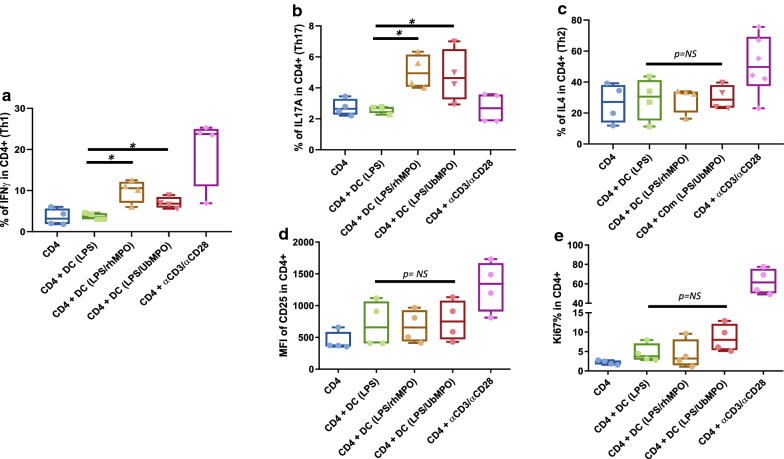
UbMPO regulates IFNγ and IL-17A production of CD4 ^+^ lymphocytes from SLE patients cocultured with LPS activated DCs with UbMPO or rhMPO. Increased production of proinflammatory cytokines, IFNγ (**a**) and IL-17A (**b**) was documented for CD4 ^+^ lymphocytes from SLE patients upon stimulation with UbMPO, meanwhile no differences were found in the IL-4 production (**c**). A differential proliferative response was found between SLE and healthy controls, with increased activation (**d**) and proliferation (**e**) (no significative) from the lupus samples and decreased proliferation from healthy controls (Additional file 1: Figure S3) towards UbMPO stimulation. *p < 0.05

**UbMPO induces IFNγ and IL-17A production as well a differential proliferation and activation profile:** By means of intracellular cytokine staining, increased production of IFNγ (Fig. 1a) and IL-17A (Fig. 1b) was found in SLE CD4^+^ T lymphocytes co-cultured with dendritic cells activated with rhMPO and UbMPO compared to the group in which the co-culture included DC activated only with LPS (*p* < 0.05). No differences were found in IL-4 synthesis (Fig. 1c) or CD25 expression (Fig. 1d). Nonetheless, a trend towards increased proliferation was observed only in the CD4^+^ lymphocytes stimulated with UbMPO/LPS in comparison with both, the lymphocytes activated with rhMPO/LPS (UbMPO/LPS vs rhMPO/LPS *p* = 0.1143) and with those activated with LPS in the absence of MPO (UbMPO/LPS vs LPS *p* = 0.1143) (Fig. 1e).

Additionally, stimulation with UbMPO was associated to lower expression of CD25 in CD4^+^ lymphocytes from healthy controls, compared to those T cells cocultured with dendritic cells activated only with rhMPO (UbMPO MFI [1751(1308–1889)] vs rhMPO MFI [2127(2042–2501] *p* < 0.05 (Additional file 1: Figure S3). Likewise, a lower proliferation (Ki67 expression) was evidenced in CD4^+^ T lymphocytes from healthy donors that were cocultured with dendritic cells stimulated with UbMPO compared to those activated with rhMPO. (UbMPO% [9.93 (8–10.3)] vs rhMPO% [11(10.5–11.73)] *p* < 0.05), (Additional file 1: Figure S3) (Additional file 1: Table S1).

**Ubiquitylation of MPO is related to HEME group modifications:** The HEME group was attached to D94 of chain α, and E242 of chain β, using an ester bond among methyl groups of catalytic site and carboxyl side chains of the subunits (Additional file 1: Figure S4, Panel A). Also, owed to its proximity to the HEME active site, the D98 was protonated, as suggested by Carpena, et al. [39]. We found three lysine: (K129-MPO, K488-MPO, and K505-MPO) as the best candidates for ubiquitylation and prepared four systems for detecting structural and conformational changes made by ubiquitylation (Additional file 1: Figure S4, Panel B).

**Contact maps at the active site:** Through the analysis of relative frequencies in the contact map of the residues close to the HEME group, with a cutoff point for bond distance < 2 Å (Additional file 1: Figure S4, Panel C); differential profiles were found depending on the ubiquitin binding site to the MPO. In this way, R239 was found near the HEME group when ubiquitin was at positions K505 or K488 (Additional file 1: Figure S4, Panel D).

**Hydration of the active site:** To investigate the dynamics of the water molecules in the active site, we measure the number of water molecules at 3 Å from the HEME group. Interestingly, ubiquitylation seemed to disrupt the amount of water in the active site (Additional file 1: Figure S4, Panels E, F, G, H). Ub-K129- and Ub-K488-MPO showed an increased amount of water molecules in the active site relative to the MPO, while Ub-K505MPO decreased the amount of water close to the HEME group. This observation could be relevant to the enzymatic activity, as the substrates are chloride ions and hydrogen peroxide, which could increase the amount of water in the active site.

**Fig. 2:**
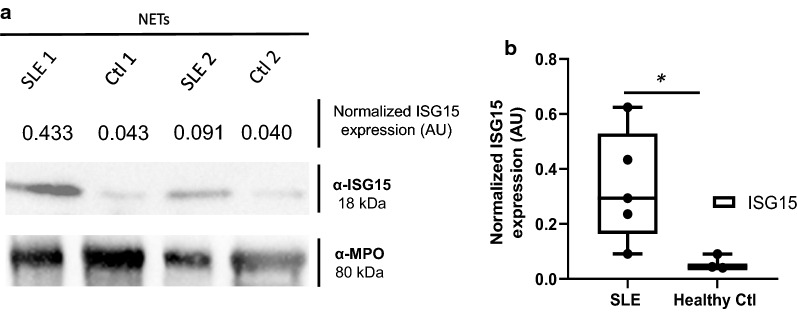
NETs from SLE patients are enriched in ISG15. **a** Western Blot analysis of LPS induced NETs from SLE patients and healthy controls (n = 2 subjects per group) was performed and quantification was done by densitometry. MPO was used as a loading control. Representative blots for ISG15 expression are included (**a**) and increased expression of ISG15 in NETs from SLE patients (n = 5) vs healthy controls (n = 3) is shown in **b**. *p < 0.05

**NETs from SLE patients contain ISG15:** We found a differential ISG15 expression characterized by higher amounts of ISG15 in NETs from active SLE patients compared with healthy donors by Western blot. (SLE [0.29(0.16–0.52)] vs healthy control ([0.04(0.040–0.090)] *p* < *0.05*) (Fig. 2a, b).

**Fig. 3:**
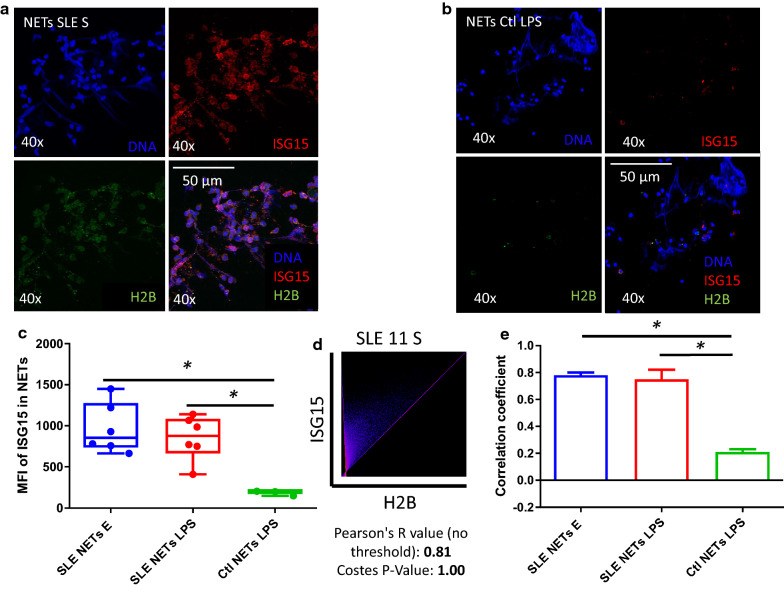
NETs from SLE patients are enriched in ISG15 and H2B is the main substrate. ISG15 and H2B expression was assessed in spontaneous and LPS induced NETs from SLE patients (n = 6) and healthy controls (n = 3) by indirect immunofluorescence (40X) and confocal microscopy. Spontaneous NETs from a representative SLE patient shows the presence of extracellular ISG15 in the NET (**a**). In contrast to the SLE sample, a representative healthy control image shows the absence of ISG15 in the NET (**b**). Blue (DNA), red (ISG15) and green (H2B). Cumulative data from SLE patients and healthy controls (n = 6 subjects per group) show increased expression of ISG15 in the SLE samples (**c**). In SLE samples, ISG15 and H2B colocalized inside NETs with a high Pearson (R = 0.81) and Costes p value (1.0) in a representative SLE patient (**d**) and boxes represent pooled data (n = 6 subjects per group) that show increased colocalization of SLE samples vs healthy controls (**e**). H2B with ISG15 had the higher colocalization R value compared to other NET proteins such as LL37 or HMGB1 (Additional file 1: Figures S5 and S6). * p < 0.05

**NETs are an extracellular source of ISG15 and colocalize with H2B in SLE:** ISG15 was found both intra and extracellularly in the NETs of patients with SLE by confocal microscopy (Fig. 3a), but not in the NETs of healthy controls (Fig. 3b). Accordingly, we found increased expression of ISG15 in lupus NETs (both spontaneous and LPS-induced) compared to healthy controls (p < 0.05) (Fig. 3c).

The colocalization analysis was performed in the area considered as a NET, by immunofluorescence for ISG15 and three potential substrates: H2B, HMGB1 or LL-37 (Additional file 1: Figures S5 and S6). We found that H2B showed the higher colocalization (r = 0.81) (Fig. 3d). The highest colocalization between ISG15 and H2B was found in spontaneous NETs of patients with SLE followed by the colocalization in LPS induced NETs from SLE patients, and as expected the lowest was observed in NETs from healthy controls, with a significant difference between lupus NETs and healthy controls samples (0.75 [0.73–0.86] vs 0.21 [0.18–0.23] *p* < 0.05) (Fig. 3e). No colocalization was found between ISG15 and HMGB1 or LL37 in NETs from healthy donor’s samples (data not shown). By this method we can confirm that there is colocalization of ISG15 and H2B in NETs of patients with SLE, but not in healthy controls, suggesting H2B as one of the proteins that interact with ISG15 in lupus patients along with NETosis as another source of ISG15 in SLE.

**Fig. 4:**
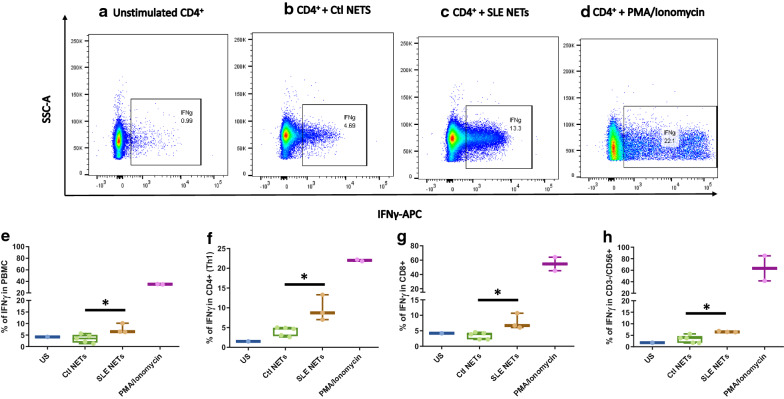
NETs contaning in ISG15 promote the production of IFNγ in different PBMCs subsets. IFNγ + cells from different PBMCs subsets from healthy controls were assessed by flow cytometry under different experimental conditions. A representative dot plot from one healthy control is shown for each experimental condition as follows: **a** Unstimulated; **b** NETs from healthy controls without ISG15 expression; **c** NETs from SLE patients that contain ISG15; **d** PMA + Ionomycin. Boxes (median ± interquartile range) represent the pooled data from healthy controls (n = 3) and show increased expression of IFNγ + cells when total PBMCs (**e**), CD4 + lymphocytes (**f**), CD8 + lymphocytes (**g**) and NK cells (**h**) were stimulated with SLE NETs enriched in ISG15. * p < 0.05

**Fig. 5:**
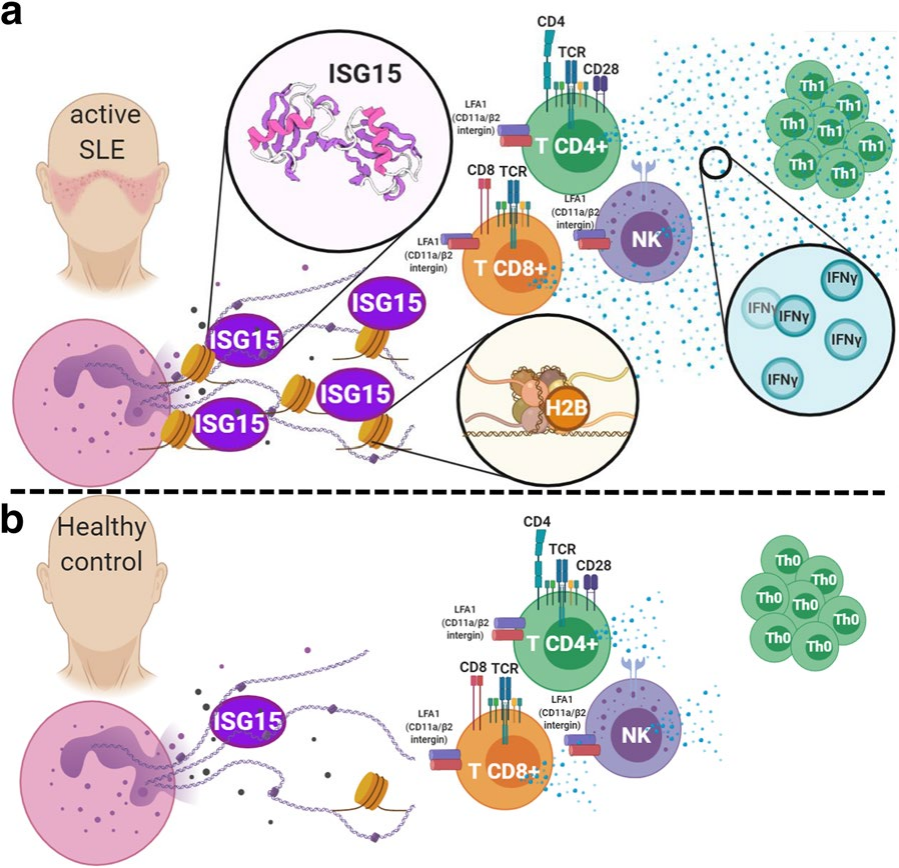
Proposed hypothetic model of NETosis as a non-canonical release mechanism of ISG15. In lupus patients, the NETs cargo is consistent with augmented amount of ISG15, which colocalized with H2B and induce the polarization of CD4, CD8 and NK to a Th1 subset that enhanced the production of IFNγ through LFA1 (CD11a/β1 integrin) (**a**). Low concentrations of ISG15 were found in healthy controls NETs with a diminished colocalization with H2B and reduction of Th1 IFNγ + CD4, CD8 and NK cells (**b**). Therefore, we proposed that ISG15 is a fingerprint of the interferogenic signature, and its release is mediated through NETosis in lupus patients

**NETs containing ISG15 from SLE patients induce IFNγ production from diverse cellular subsets:** To address the functional impact of ISG15 on the NETs from lupus patients, the production of IFNγ in subsets of T lymphocytes (CD4^+^ and CD8^+^) and NK cells was evaluated by multiparametric flow cytometry. Higher IFNγ production was found in total PBMCs that were stimulated with NETs from lupus patients that previously had shown to contain ISG15 (Fig. 4b) compared to cells stimulated with NETs from healthy controls (Fig. 4c) (%IFNγ 6.5[6.31–10.1] vs 3.5[1.7–5.1]) *p* < 0.05). Figure 5 shows a higher production of IFNγ by stimulating different subsets of PBMCs with ISG15 containing NETs from SLE patients, compared with NETs of healthy donors, lacking such post-translational modification, including CD4^+^ lymphocytes (Fig. 4f), CD8^+^ lymphocytes (Fig. 4g), and NK CD56^+^ lymphocytes (Fig. 4h).

## Discussion

In the present work, we demonstrated the presence of PTMs related to ubiquitin tag (total ubiquitin and ISG15), in NETs from SLE patients, and their impact in the reactivation, proliferation and polarization of CD4^+^ cells of SLE patients and controls. Furthermore, to our knowledge this is the first report on the differential patterns of water molecules and conformational changes in residues near the catalytic site of MPO conjugated to ubiquitin. Interestingly, our data also suggest NETs as a source of ISG15 in SLE, shows that H2B interact with this PTM and a potential role in regulating IFNγ secretion by T lymphocytes in SLE.

The trend towards increased proliferative responses of CD4^+^ T lymphocytes from patients with SLE, as well as the polarization towards Th1 and Th17 subpopulations producing IFNγ or IL-17A in the presence of components of NETs such as recombinant MPO and ubiquitylated MPO is consistent with a previous study in which CD4^+^ and CD8^+^ T lymphocytes from controls stimulated with supernatants of NETs induced in vitro, had higher levels of CD25, CD69, phosphorylation of ZAP70, secretion of IFNγ and IL-17A; however, there was no increase in the percentage of proliferation with ethinyl deoxyuridine (EdU) [40]. The difference described between patients and controls in CD25 or Ki67% could be related to a differential threshold for activation and proliferation signals, which could be reached by some of the NET components and therefore able to induce the production of effector cytokines such as IFNγ and IL-17A, but insufficient to fully activate T cell proliferation. In agreement to this, other studies have described proliferation of PBMCs from patients with vasculitis associated with anti-neutrophil cytoplasm antibody (ANCA) by increasing MPO concentrations to 10 μg/mL [7, 41], which corroborates the requirement for a high concentration of MPO for the regulation of proliferation in T lymphocytes. Furthermore, the ability to produce IFNγ by PBMCs from patients with ANCA vasculitis at the dose we used for the activation of dendritic cells (5 μg/mL), had been previously described by ELISA and this production was not carried out when using lower doses (0.5 μg/mL) [8]. This is the first study addressing the biological effect of the ubiquitin tag in MPO in the context of systemic autoimmunity. Interestingly, the proliferative response of CD4^+^ lymphocytes depends on the ubiquitin tag as well as the disease state (ie healthy controls vs SLE). It has been described that the type of response that is mounted (proinflammatory vs anti-inflammatory) is mainly related to the context in which the antigenic determinant (immunogen or tolerogen) is seen, highlighting the microenvironment, the PTMs and the disease state as key elements for the functional effect of every antigen [42]. In patients with anti-MPO glomerulonephritis, MPO-specific CD4^+^ T cells mediate organ damage, recognizing MPO released by NETosis as an autoantigen, conducting delayed-type hypersensitivity promoting IFN-γ and IL-17A production [43]. Accordingly, we have shown that SLE patients have high titers of anti-MPO antibodies, and higher titers of anti-UbMPO [11]. However, in other scenario were endogenous MPO is not an autoantigen (like collagen-induced arthritis, pristine-induced lupus nephritis or healthy donors) MPO attenuates or even suppresses T cells responses, including decreased proliferation of human T cells in vitro [44], lower DC activation and migration, showing an increased proliferation, activation and proinflammatory cytokine production in MPO^−/−^ mice [6]. This differentiation of responses may depend of unusual mechanisms driven by MPO [45], making clear that the context in which the immune response is mounted, matters.

It is feasible that the relationship of ubiquitin with a lower activation and proliferation of CD4^+^ T cells in controls is due to its ability to mask potentially dangerous antigens for the system (such as MPO in NETs) [46]. Nonetheless, this relationship is in turn unbalanced in the context of SLE where both MPO with and without ubiquitin was able to induce polarization of CD4^+^ T cells towards a Th1 and Th17 effector phenotype. This is in agreement with previous data from our research group suggesting that lupus patients have a ubiquitin deficiency state involving diverse cellular subsets and processes, in which they express lower amounts of E3 ligases (key enzymes for ubiquitylation) in T cells [47, 48] as well as diminished polyubiquitin K63 chains in NETs [11], both with functional impact, such as resistance to anergy and altered macrophage responses, respectively. Accordingly, in healthy controls, the presence of ubiquitin tag involves a suppressive mechanism when it is bound to a molecule with a dual role in inflammatory signaling, such as MPO, which might be diminished in the context of systemic autoimmune diseases, such as SLE, explaining the shift towards Th1 and Th17 in CD4^+^ cells in patients and the tolerogenic role in controls, diminishing the activation and proliferative responses.

The molecular dynamics simulations allowed to describe the closeness of arginine 239 (R239) when ubiquitin was bound to K505 or K488 residues, compared to native MPO. R239 is an important residue of the polypeptide chain lying near the distal face of the HEME group [49] and has been involved in the catalytic mechanism of MPO [50]. There is evidence using molecular docking that the LGM2605 peptide, by its hydroxyl group, inhibits the enzymatic activity of MPO by blocking R239. The authors propose that while this position may not directly displace H_2_O_2_ binding, blocking the substrate channel may slow H_2_O_2_ access to HEME [51]. Our results using contact map analysis allowed us to find that R239 decreased its distance from the HEME group when ubiquitin was conjugated to K505 or K488 compared to native MPO.

One of the most relevant changes in the structural dynamics of the MPO was the differential hydration of the active site mediated by the ubiquitinated lysine residue. Water molecules are relevant to receptor-ligand recognition because they modify the geometry of the active site and contribute to binding affinity. Our results propose that when ubiquitin conjugates to K129 or K488, the number of water molecules close to the HEME group increases, and the opposite occurs when ubiquitin binds to K505.

Together, the closeness of R239 and the increased amount of internal water molecules in the active site; implies a higher probability of entry to the active site by the reaction substrates (Cl^−^ and H_2_O_2_), and therefore may increase the production of reactive intermediates such as taurine chloramine, implying amplified peroxidase activity. Taurine chloramine is an inhibitory mechanism of activation and proliferation that diminish the release of IL-12 and TNFα from DC and subsequently IL-2 and IL-10 from T cells in vitro [52].

Furthermore, the evolution of structures is consistent with functional specificity, and ubiquitin-like proteins are an obvious example of the pleiotropism inherent in their function, as is the case with the 15 kDa IFN-related gene. ISG15 is another PTM that has a great diversity of functions depending on its location. Recently it has been identified the lymphocyte function-associated antigen 1 (LFA-1) or CD11a/β1 integrin as the putative receptor of ISG15 in NK cells inducing the activation of Src kinases and IFNγ and IL-10 synthesis [52]. However, currently, the release mechanism is unknown, secretory granules of neutrophils, lysosomes, exosomes, apoptotic bodies and microvesicles released from infected macrophages have been suggested as probable mechanisms [53]. Based on our results, we can propose a new non-canonical secretion mechanism of ISG15 by NETosis in patients with SLE. To our knowledge, this is the first report of extracellular ISG15 as a component of NETs only in SLE patients, highlighting its role in this disease. It is feasible to propose NETosis as one of the non-canonical secretion mechanisms of extracellular ISG15, since the increased levels of type I IFN, and the molecular transcriptional signature of SLE are suggested to be key pathogenic players in the disease [14, 54].

In cells that respond to interferon, free extracellular ISG15 has a "cytokine-like" activity with evidence supporting its ability to induce the production of IFNγ [55, 56]. Additionally, controversy exists about the pathogenic role of ISG15, since the overexpression of ISG15 in NETs of SLE patients could be both a consequence of the overstimulation by the IFNα/β signature, and a signal from NETs involved in the production of IFNγ by lymphocyte subpopulations [13, 57]. Our data show that indeed NETs from SLE patients are capable of inducing IFNγ production in CD4^+^ T lymphocytes as has been previously shown by Tillack KB, et al. [40].

Finally, we show that the protein cargo of NETs from SLE patients contain ISG15 and able to induce IFNγ production, which agrees with previous data by Iglesias-Guimarais, et al. [58] which show that in addition to CD4^+^ lymphocytes, IFNγ production was also reported by other subpopulations such as CD8^+^ and NK cells, both known targets of action of extracellular ISG15 [58], which is also produced by lupus plasma cells [57]. Therefore, it can be argued that NETs promote the activation of these cells with the subsequent production of IFNγ in response (among other stimuli) to free ISG15 [59, 60]. Thus, we propose ISG15 in NETs as a new component in the physiopathogenic scheme of autoimmune diseases such as SLE, with an effector correlate that includes the induction of Th1 lymphocytes with a proinflammatory potential (Fig. 5, Proposed hypothetic model). There is evidence that the deficiency of ISG15, through the ISGylation of USP18, can degrade this potent inhibitor of IFNα/β-mediated signaling and promote its accumulation [12], therefore ISG15 has even been proposed as a down regulator of type 1 IFN [59], and may have a different functional profile depending on its interactions to another extracellular proteins or its release, as shown in the present study and previous reports in which the administration of free ISG15 as an adjuvant, helps to enhance the production of IFNγ and an increase in the cytolytic activity of CD8^+^ T cells against papillomavirus [61].

More than 30% of the ISG15 targets were described by mass spectrometry as nuclear proteins [13]. These data suggest histones (nuclear proteins that are part of NETs) as potential targets for this PTM [62, 63]. The ubiquitin label in H2B (K123) has been found to affect chromatin dynamics and RNA polymerase passage to facilitate a robust transcription system [64, 65]. So far, this has been the closest evidence that ubiquitin or some other ubiquitin-like protein is related to histone interaction, specifically H2B. This contact might induce changes in structure and function as previously reported with other PTMs in chromatin, and a source of autoantigens in autoimmune pathologies [63]. However, it had been described that intracellular ISGylation inhibits the secretion of ISG15, so it seems that that they have separated pathways, one inhibiting the other. Novel de-ISGylases can reverse intracellular ISGylation improving the extracellular ISG15 secretion [66]. Consequently, is more plausible that ISG15 coming from lupus NETs is in a free, extracellularly, cytokine-like form accounting, at least in part, for durable pro-inflammatory responses. Subsequent studies will be needed to demonstrate this issue.

Our study has many limitations. First, it is a transversal study with no tracing of patient’s characteristics. Also, we used effector CD4^+^ in the cocultures with DC, instead of naïve CD4^+^, resulting in a reactivation profile of ex vivo Th1 or Th17 instead of in vitro Th0 polarization. Nevertheless, it is the first study to address the in vitro*, *in silico and ex vivo role of ubiquitin and NETosis-released-ISG15, their impact on cellular responses from SLE patients as well as the improvement in our knowledge of the pathophysiology of lupus and autoimmunity.

## Conclusions

In summary, UbMPO can induce the reactivation of Th1 and Th17 cells, producing IFNγ and IL-17A; enhanced proliferation in SLE patients, as well as dampened activation and proliferation in healthy donors T cells, highlighting the presence of changes in the intrinsic dynamics of MPO when conjugated with ubiquitin. Besides, our data suggest that NETosis is a non-canonical release mechanism for ISG15, suggest H2B as a probable substrate for ISG15 in SLE patients and these ISG15-containing NETs from SLE patients promote enhanced production of IFNγ, implying a new field in pleiotropic functions of PTM promoting tolerogenic and immunogenic responses during autoimmune diseases.

## Supplementary information

**Additional file 1: Figure S1.** Western Blot of total anti-ubiquitin and controls used for anti-ISG15 antibody. A lysate of HeLa cells, recombinant human MPO (rhMPO) are observed, both without the presence of ubiquitin and MPO with ubiquitin as negative controls of the reaction (since all the substrates necessary for the enzymatic reaction are added except ATP); in the last lane the product of the complete in vitro ubiquitylation reaction of the MPO (Panel A). A lysate of transfected human HEK293T cells that overexpressed ISG15 used as a positive control and empty vector transfected control cell lysate (HEK293) as a negative control for Western Blot experiments of the Fig. 2 (Panel B). **Figure S2.** Representative dot plot of the gating strategy for the assessment of the percentage of CD25, Ki67, IFNγ. The lymphocyte population was selected from a graph of size and complexity (Panel A). Then the singlets were obtained by comparing area granularity against height granularity (Panel B). Viable cells were determined using FVS700 exclusion staining (Panel C). This subpopulation of lymphocytes was determined by CD4^+^ staining (Panel D). Subsequently, activation markers were determined in this subpopulation (CD25^+^ in Panel E), proliferation (Ki67^+^ in Panel F) and activation through the production of cytokines (IFNγ^+^ in panel G). **Figure S3.** UbMPO diminish CD4^+^ lymphocyte activation and proliferation of healthy controls were cocultured with LPS activated DCs with UbMPO. No differences were found in the production of cytokines among groups of interest (A-C). However, lower activation of healthy control determined by CD25 expression in CD4^+^ lymphocytes upon UbMPO stimulation was found compared with rhMPO (D). Decreased proliferation from healthy controls towards UbMPO stimulation (E). * p < 0.05. **Figure S4.** Molecular dynamics simulations MPO active site and ubiquitylation sites. Close-up of the HEME group in the native MPO active site showing ester bonds of the D94, E292 side chains and the methyls at position 3 and 13 of the HEME group (A). Representation in licorice. Fragments of chain a and chain b are shown in orange and ochre ribbons as context of the active site, as well as H. Structure of the native MPO in cartoons representation (B), chain a in magenta and chain b in ice blue. In licorice representation the HEME group, axial histidine, lysines 129, 488 and 505, and three water molecules in the active site. Color code: Color code C, cyan, O, red, H, white N, blue, Fe, pink. Visual representation of the residues near the HEME. R239 was found close to the active site only when ubiquitin was conjugated to K505 or K488 (C). Contact map of the residues of the MPO in close contact with the HEME group, highlights the differential profile when the ubiquitin is found in K505 or K488 over R239 (D). Number of water molecules in the active site as a function of simulation time. In the crystal structure, there are 3.5 water molecules in this region of the protein. Compared to the native MPO (E), ubiquitylation at K129 (F) or K488 (G) the number of water molecules increases by 1 to 3 molecules; in K505 (H) the amount of water in the active decreases up to 1. The hydration level at the active site was sensitive to the ubiquitylation site. **Figure S5.** Colocalization of LL37 with ISG15 in lupus NETs. ISG15 and known proteins expressed in NETs such as LL37, was assessed in spontaneous NETs from SLE patient by indirect immunofluorescence (40X) and confocal microscopy. In a representative SLE patient, ISG15 and LL37 colocalized inside NETs with a lower Pearson (R = 0.40) and Costes p value (1.0), compared with colocalization of ISG15 and H2B (Fig. 4). Blue (DNA), red (ISG15) and green (LL37). **Figure S6.** Colocalization of HMGB1 with ISG15 in lupus NETs. ISG15 and known proteins expressed in NETs such as HMGB1, was assessed in spontaneous NETs from SLE patient by indirect immunofluorescence (40X) and confocal microscopy. In a representative SLE patient, ISG15 and HMGB1 colocalized inside NETs with a lower Pearson (R = 0.57) and Costes p value (1.0), compared with colocalization of ISG15 and H2B (Fig. 4). Blue (DNA), red (ISG15) and green (HMGB1). **Table S1.** Expression of activation, proliferation markers and cytokines in co-coltures of CD4^+^ with DC from SLE patients and healthy controls. Relative frequency and mean fluorescence intensity of activation markers such as CD25, Tregs (CD4 + /CD25 + /FoxP3 +), CD83, HLA-DR. Proliferation measured by Ki67% and production of IFNγ, IL-17A, IL-4. Statistically differences among interest groups are highlighted in red (UbMPO/LPS and rhMPO/LPS vs LPS). DCs: dendritic cells. IQR = interquartile range. LPS = lipopolysaccharide. rhMPO = recombinant human myeloperoxidase. UbMPO = Ubiquitylated myeloperoxidase.

## Data Availability

The datasets used and/or analyzed during the current study are available from the corresponding author on reasonable request.

## Abbreviations

ACR: American College of Rheumatology
ANCA: Antineutrophil cytoplasmic antibodies
CHARMM: Chemistry at HARvard Macromolecular Mechanics
Cl^−^: Chloride
DC: Dendritic cells
dsDNA: Double stranded deoxyribonucleic acid
ECL: Enhanced chemiluminescence
EdU: Ethinyl deoxyuridine
ELISA: Enzyme-linked immunosorbent assay
FBS: Fetal bovine serum
FoxP3: Forkhead box protein P3
GM-CSF: Granulocyte macrophage colony-stimulating factor
H2B: Histone 2 B
H_2_O_2_: Hydrogen peroxide
HEPES: 4-(2-Hydroxyethyl)-1-piperazineethanesulfonic acid
HMGB1: High mobility group box 1 protein
HRP: Horseradish peroxidase
IFN: Interferon
IIWM: Internal water molecules
IQR: Interquartile range
ISG15: Interferon-stimulated gene 15
K129-MPO: Lysine129 of myeloperoxidase
K488-MPO: Lysine488 of myeloperoxidase
K505-MPO: Lysine505 of myeloperoxidase
LFA-1: Lymphocyte function-associated antigen 1
LGM2605: Synthetic secoisolariciresinol diglucoside
LL37: Cathelicidin antimicrobial peptide 18
LPS: Lipopolysaccharide
MACS: Magnetic cell separation
MoDCs: Monocyte derived dendritic cells
MPO: Myeloperoxidase
NaCl: Sodium chloride
NAMD: Nanoscale molecular dynamics
NE: Neutrophil elastase
NETs: Neutrophil extracellular traps
PBMCs: Peripheral blood mononuclear cells
PBS: Phosphate buffered saline
PDB: Protein data bank
PMA: Phorbol 12-myristate 13-acetate
PTM: Post-translational modification
PVDF: Polyvinylidene fluoride
R239: Arginine 239
rhMPO: Recombinant human MPO
RMSD: Root-mean-square deviation
RNA: Ribonucleic acid
ROS: Reactive oxygen species
RPMI: Roswell Park Memorial Institute
SASA: Solvent-accessible surface area
SLE: Systemic lupus erythematosus
SLEDAI: Systemic lupus erythematosus disease activity index
TBS: Tris-buffered saline
TNF-α: Tumor necrosis factor alpha
UbMPO: Ubiquitylated MPO
USP18: Ubiquitin specific peptidase 18
VMD: Visual molecular dynamics
ZAP70: Zeta chain of T cell receptor associated protein kinase 70
