# Supplementary material for: Conformational changes in myeloperoxidase induced by ubiquitin and NETs containing free ISG15 from systemic lupus erythematosus patients promote a pro-inflammatory cytokine response in CD4+ T cells
Source: J Transl Med. 2020 Nov 11;18:429. doi: 10.1186/s12967-020-02604-5 (PMC7659105; doi:10.1186/s12967-020-02604-5)
Supplement: Supplementary file 1 — Additional file 1: Figure S1. Western Blot of total anti-ubiquitin and controls used for anti-ISG15 antibody. A lysate of HeLa cells, recombinant human MPO (rhMPO) are observed, both without the presence of ubiquitin and MPO with ubiquitin as negative controls of the reaction (since all the substrates necessary for the enzymatic reaction are added except ATP); in the last lane the product of the complete in vitro ubiquitylation reaction of the MPO (Panel A). A lysate of transfected human HEK293T cells that overexpressed ISG15 used as a positive control and empty vector transfected control cell lysate (HEK293) as a negative control for Western Blot experiments of the Fig. 2 (Panel B). Figure S2. Representative dot plot of the gating strategy for the assessment of the percentage of CD25, Ki67, IFNγ. The lymphocyte population was selected from a graph of size and complexity (Panel A). Then the singlets were obtained by comparing area granularity against height granularity (Panel B). Viable cells were determined using FVS700 exclusion staining (Panel C). This subpopulation of lymphocytes was determined by CD4+ staining (Panel D). Subsequently, activation markers were determined in this subpopulation (CD25+ in Panel E), proliferation (Ki67+ in Panel F) and activation through the production of cytokines (IFNγ+ in panel G). Figure S3. UbMPO diminish CD4+ lymphocyte activation and proliferation of healthy controls were cocultured with LPS activated DCs with UbMPO. No differences were found in the production of cytokines among groups of interest (A-C). However, lower activation of healthy control determined by CD25 expression in CD4+ lymphocytes upon UbMPO stimulation was found compared with rhMPO (D). Decreased proliferation from healthy controls towards UbMPO stimulation (E). * p < 0.05. Figure S4. Molecular dynamics simulations MPO active site and ubiquitylation sites. Close-up of the HEME group in the native MPO active site showing ester bonds of the D94, E292 side chain [file 12967_2020_2604_MOESM1_ESM.pptx]

## Slide 1
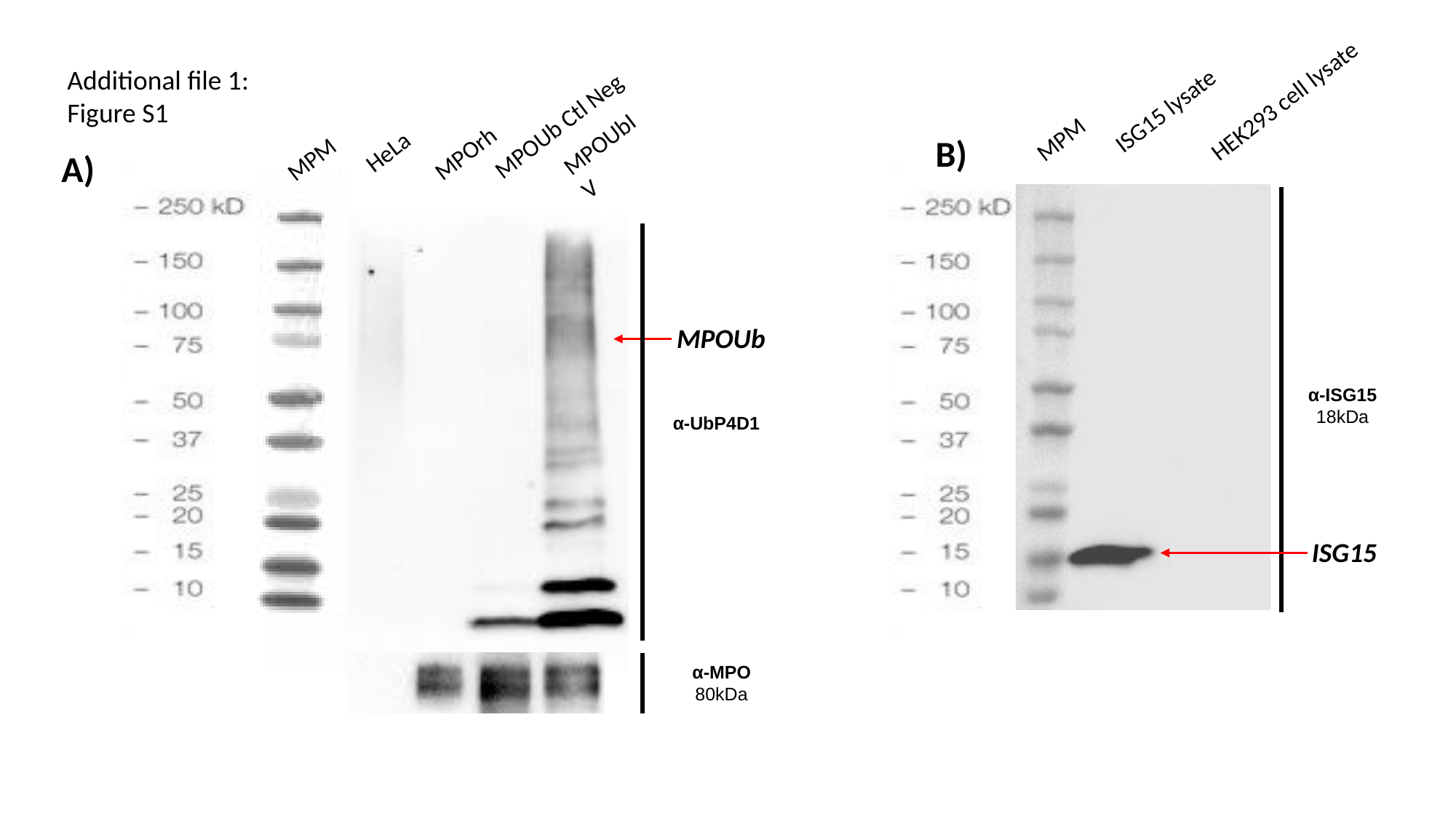

HEK293 cell lysate
MPOUb Ctl Neg
ISG15 lysate
Additional file 1: Figure S1
MPM
MPOUbIV
HeLa
MPOrh
MPM
B)
A)
MPOUb
α-ISG15
18kDa
α-UbP4D1
ISG15
α-MPO
80kDa

## Slide 2
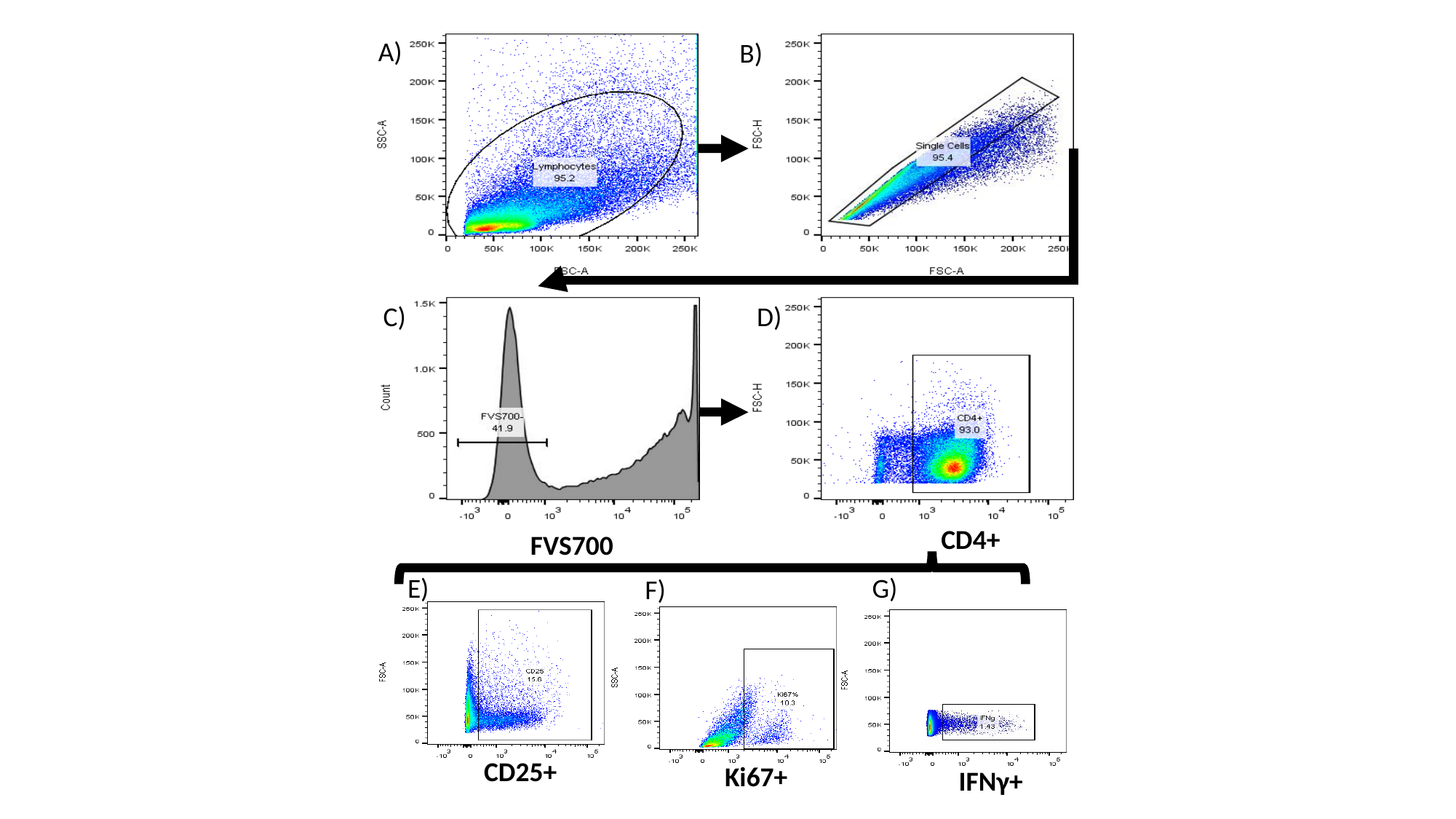

A)
B)
C)
D)
FVS700
CD4+
E)
G)
F)
CD25+
Ki67+
IFNγ+
Additional file 1: Figure S2

## Slide 3
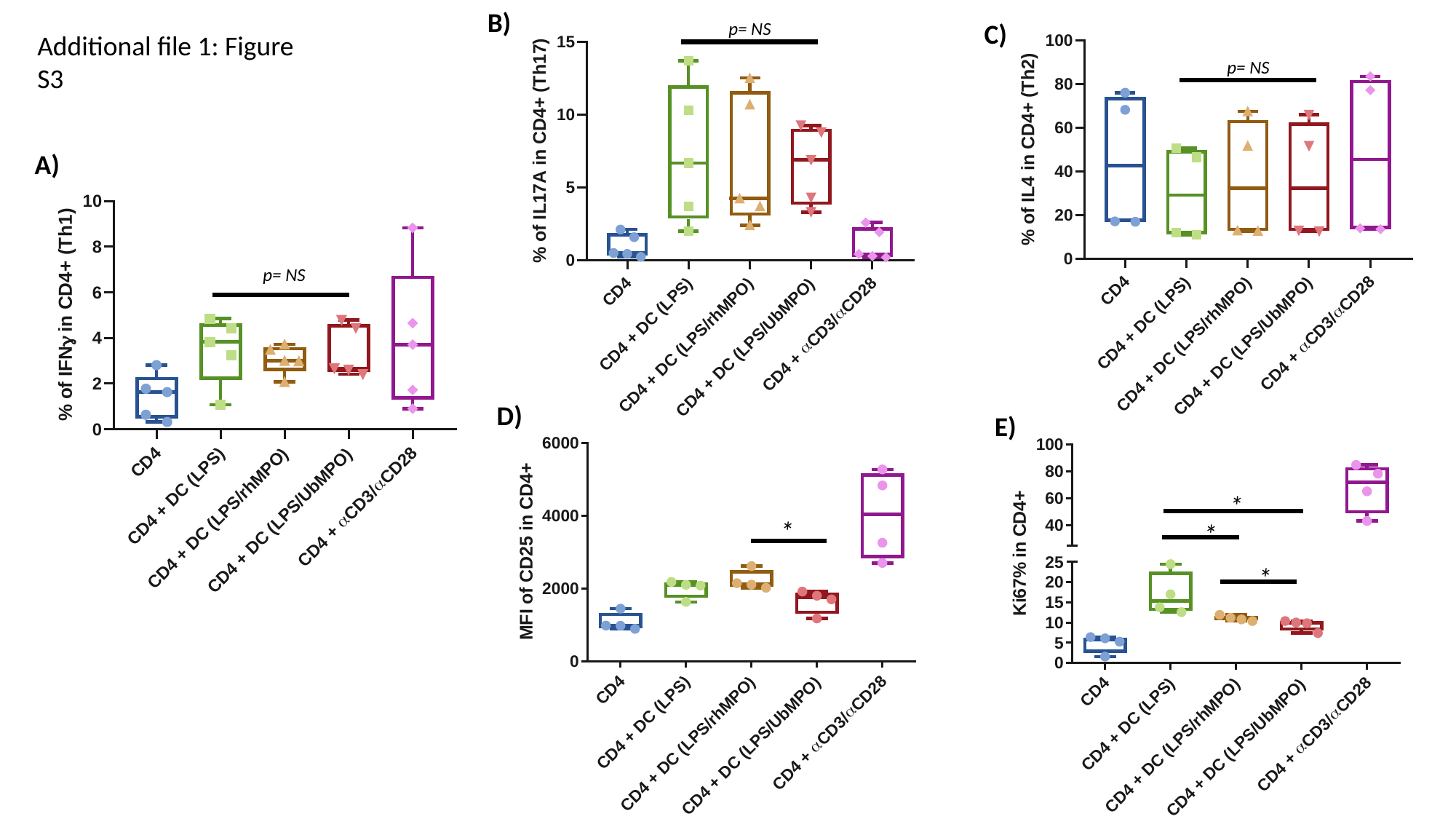

B)
C)
p= NS
p= NS
Additional file 1: Figure S3
A)
p= NS
D)
*
E)
*
*
*

## Slide 4
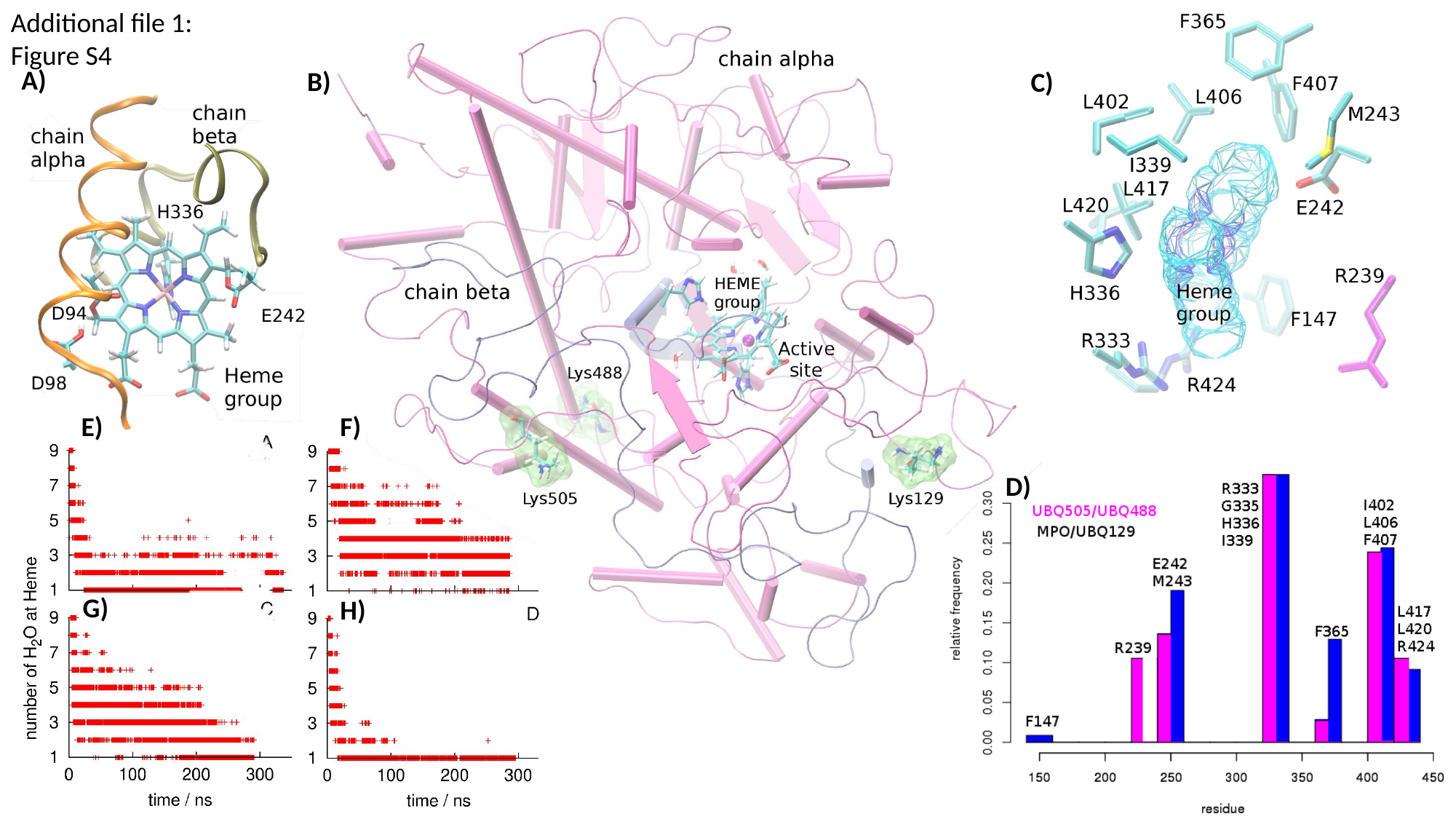

Additional file 1: Figure S4
A)
B)
C)
E)
F)
D)
G)
H)

## Slide 5
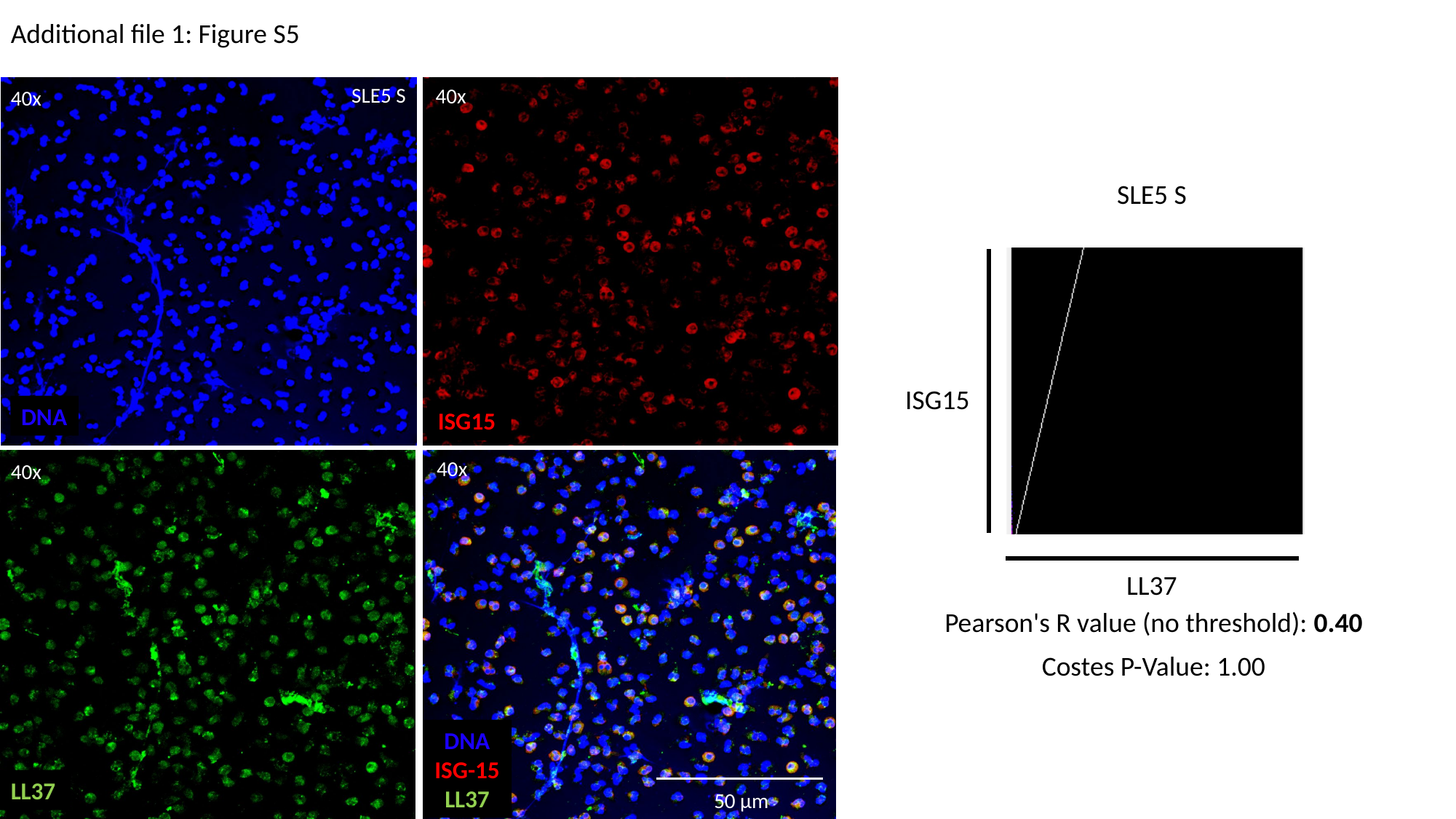

Additional file 1: Figure S5
SLE5 S
40x
40x
SLE5 S
ISG15
DNA
ISG15
40x
40x
LL37
Pearson's R value (no threshold): 0.40
Costes P-Value: 1.00
DNA
ISG-15
LL37
LL37
50 μm

## Slide 6
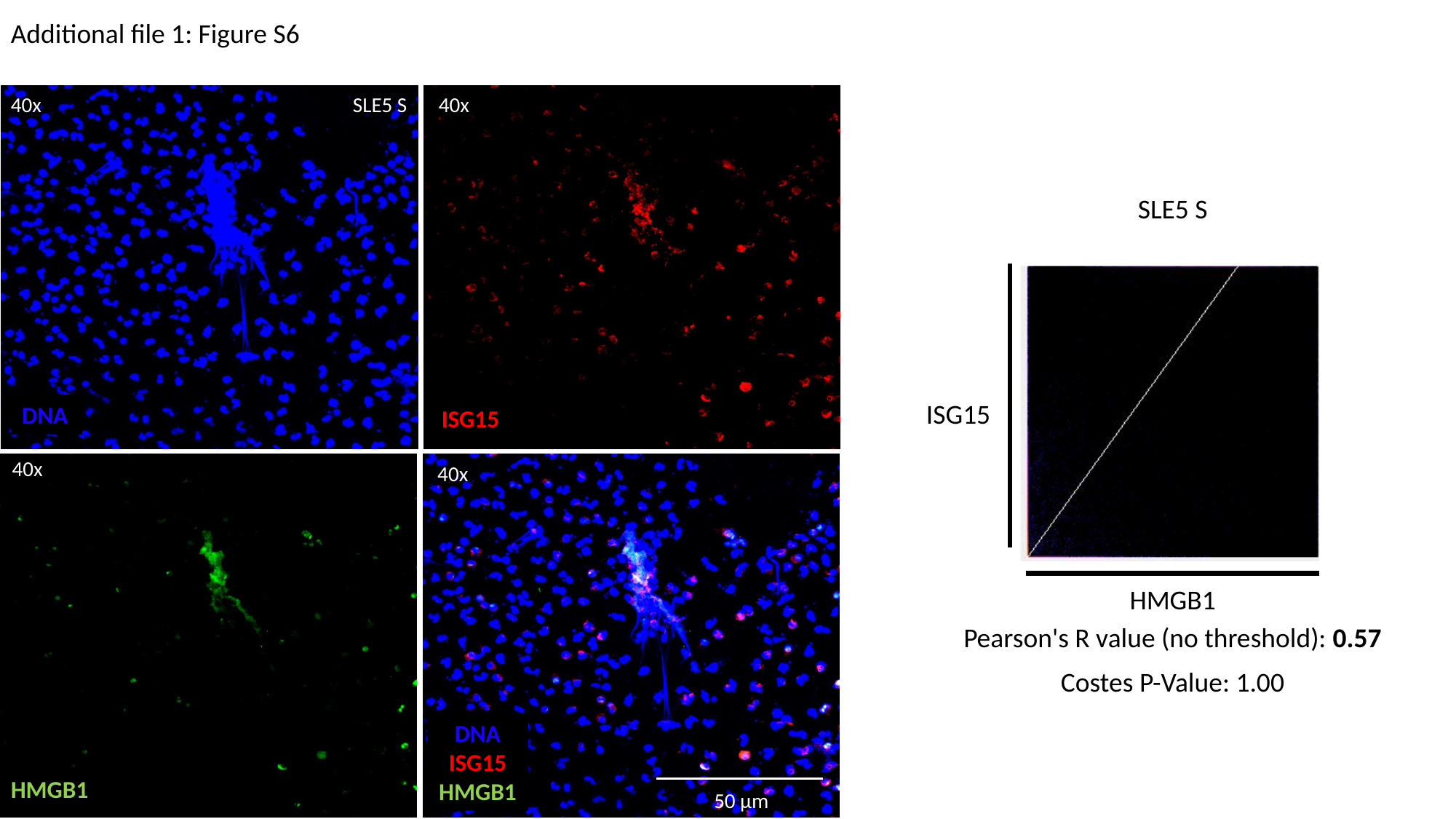

Additional file 1: Figure S6
40x
SLE5 S
40x
SLE5 S
ISG15
DNA
ISG15
40x
40x
HMGB1
Pearson's R value (no threshold): 0.57
Costes P-Value: 1.00
DNA
ISG15
HMGB1
HMGB1
50 μm

## Slide 7
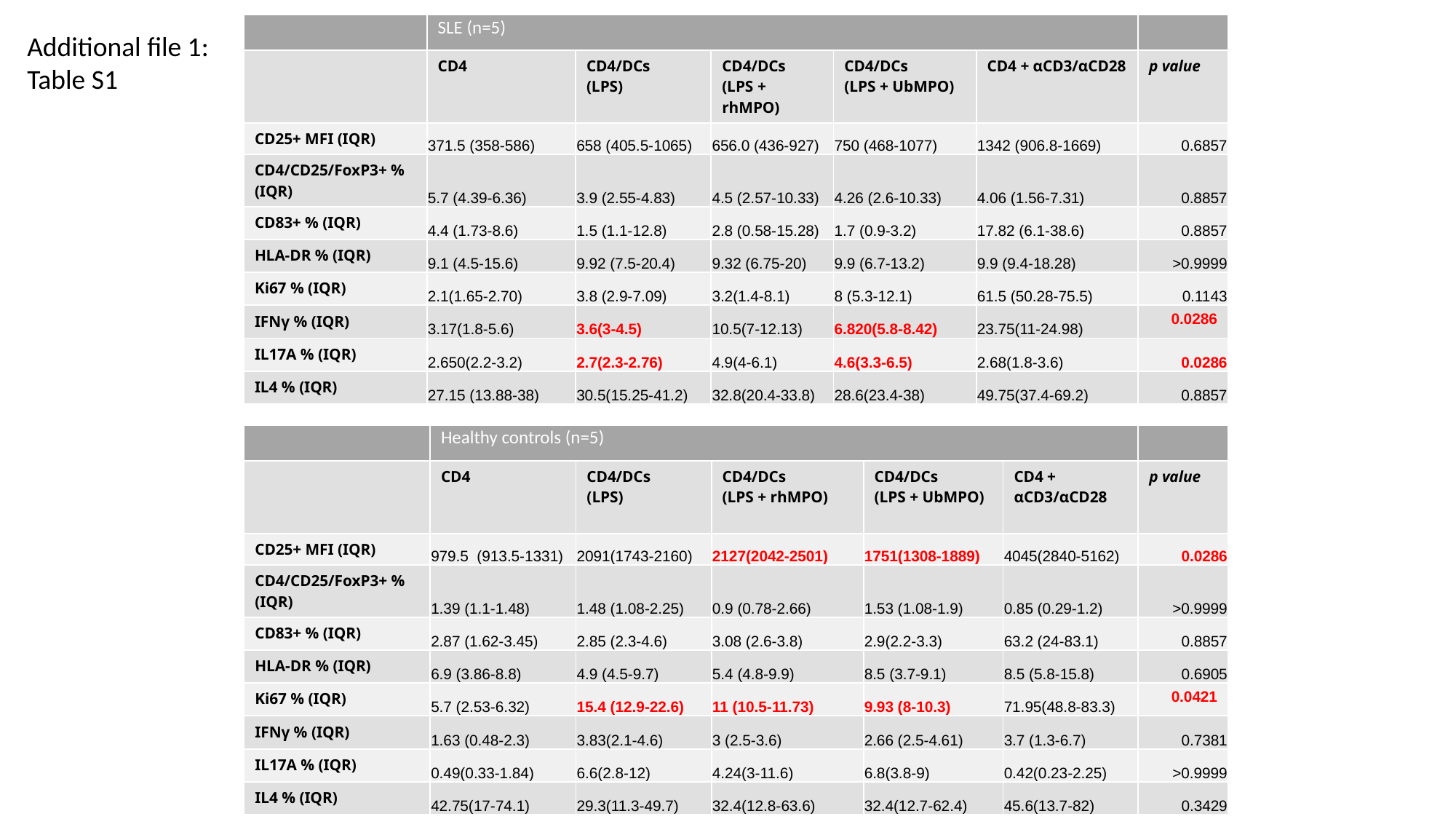

| | SLE (n=5) | | | | | |
| --- | --- | --- | --- | --- | --- | --- |
| | CD4 | CD4/DCs (LPS) | CD4/DCs (LPS + rhMPO) | CD4/DCs (LPS + UbMPO) | CD4 + αCD3/αCD28 | p value |
| CD25+ MFI (IQR) | 371.5 (358-586) | 658 (405.5-1065) | 656.0 (436-927) | 750 (468-1077) | 1342 (906.8-1669) | 0.6857 |
| CD4/CD25/FoxP3+ % (IQR) | 5.7 (4.39-6.36) | 3.9 (2.55-4.83) | 4.5 (2.57-10.33) | 4.26 (2.6-10.33) | 4.06 (1.56-7.31) | 0.8857 |
| CD83+ % (IQR) | 4.4 (1.73-8.6) | 1.5 (1.1-12.8) | 2.8 (0.58-15.28) | 1.7 (0.9-3.2) | 17.82 (6.1-38.6) | 0.8857 |
| HLA-DR % (IQR) | 9.1 (4.5-15.6) | 9.92 (7.5-20.4) | 9.32 (6.75-20) | 9.9 (6.7-13.2) | 9.9 (9.4-18.28) | >0.9999 |
| Ki67 % (IQR) | 2.1(1.65-2.70) | 3.8 (2.9-7.09) | 3.2(1.4-8.1) | 8 (5.3-12.1) | 61.5 (50.28-75.5) | 0.1143 |
| IFNγ % (IQR) | 3.17(1.8-5.6) | 3.6(3-4.5) | 10.5(7-12.13) | 6.820(5.8-8.42) | 23.75(11-24.98) | 0.0286 |
| IL17A % (IQR) | 2.650(2.2-3.2) | 2.7(2.3-2.76) | 4.9(4-6.1) | 4.6(3.3-6.5) | 2.68(1.8-3.6) | 0.0286 |
| IL4 % (IQR) | 27.15 (13.88-38) | 30.5(15.25-41.2) | 32.8(20.4-33.8) | 28.6(23.4-38) | 49.75(37.4-69.2) | 0.8857 |
Additional file 1: Table S1
| | Healthy controls (n=5) | | | | | |
| --- | --- | --- | --- | --- | --- | --- |
| | CD4 | CD4/DCs (LPS) | CD4/DCs (LPS + rhMPO) | CD4/DCs (LPS + UbMPO) | CD4 + αCD3/αCD28 | p value |
| CD25+ MFI (IQR) | 979.5 (913.5-1331) | 2091(1743-2160) | 2127(2042-2501) | 1751(1308-1889) | 4045(2840-5162) | 0.0286 |
| CD4/CD25/FoxP3+ % (IQR) | 1.39 (1.1-1.48) | 1.48 (1.08-2.25) | 0.9 (0.78-2.66) | 1.53 (1.08-1.9) | 0.85 (0.29-1.2) | >0.9999 |
| CD83+ % (IQR) | 2.87 (1.62-3.45) | 2.85 (2.3-4.6) | 3.08 (2.6-3.8) | 2.9(2.2-3.3) | 63.2 (24-83.1) | 0.8857 |
| HLA-DR % (IQR) | 6.9 (3.86-8.8) | 4.9 (4.5-9.7) | 5.4 (4.8-9.9) | 8.5 (3.7-9.1) | 8.5 (5.8-15.8) | 0.6905 |
| Ki67 % (IQR) | 5.7 (2.53-6.32) | 15.4 (12.9-22.6) | 11 (10.5-11.73) | 9.93 (8-10.3) | 71.95(48.8-83.3) | 0.0421 |
| IFNγ % (IQR) | 1.63 (0.48-2.3) | 3.83(2.1-4.6) | 3 (2.5-3.6) | 2.66 (2.5-4.61) | 3.7 (1.3-6.7) | 0.7381 |
| IL17A % (IQR) | 0.49(0.33-1.84) | 6.6(2.8-12) | 4.24(3-11.6) | 6.8(3.8-9) | 0.42(0.23-2.25) | >0.9999 |
| IL4 % (IQR) | 42.75(17-74.1) | 29.3(11.3-49.7) | 32.4(12.8-63.6) | 32.4(12.7-62.4) | 45.6(13.7-82) | 0.3429 |
